# Circulating MicroRNAs predict glycemic improvement and response to a behavioral intervention

**DOI:** 10.1186/s40364-021-00317-5

**Published:** 2021-08-23

**Authors:** Elena Flowers, Isabel Elaine Allen, Alka M. Kanaya, Bradley E. Aouizerat

**Affiliations:** 1 Department of Physiological Nursing, University of California, San Francisco, 2 Koret Way, #605L, CA 94143-0610 San Francisco, USA; 2 Institute for Human Genetics, University of California, San Francisco, 2 Koret Way, #605L, CA 94143-0610 San Francisco, USA; 3 Department of Epidemiology and Biostatistics, University of California, San Francisco, USA; 4 Department of Medicine, University of California, San Francisco, USA; 5 Bluestone Center for Clinical Research, New York University, New York, USA; 6 Department of Oral and Maxillofacial Surgery, New York University, New York, USA

**Keywords:** microRNA, Diabetes, Fasting blood glucose, Biomarker, Yoga

## Abstract

**Background:**

MicroRNAs may be important regulators of risk for type 2 diabetes. The purpose of this longitudinal observational study was to assess whether circulating microRNAs predicted improvements in fasting blood glucose, a major risk factor for type 2 diabetes, over 12 months.

**Methods:**

The study included participants (n = 82) from a previously completed trial that tested the effect of restorative yoga on individuals with prediabetes. Circulating microRNAs were measured using a flow cytometry miRNA assay. Linear models were used to determine the optimal sets of microRNA predictors overall and by intervention group.

**Results:**

Subsets of microRNAs were significant predictors of final fasting blood glucose after 12-months (*R*^2^ = 0.754,* p* < 0.001) and changes in fasting blood glucose over 12-months (*R*^2^ = 0.731, *p* < 0.001). Three microRNAs (let-7c, miR-363, miR-374b) were significant for the control group only, however there was no significant interaction by intervention group.

**Conclusions:**

Circulating microRNAs are significant predictors of fasting blood glucose in individuals with prediabetes. Among the identified microRNAs, several have previously been associated with risk for type 2 diabetes. This is one of the first studies to use a longitudinal design to assess whether microRNAs predict changes in fasting blood glucose over time. Further exploration of the function of the microRNAs included in these models may provide new insights about the complex etiology of type 2 diabetes and responses to behavioral risk reduction interventions.

**Trial registration:**

This study was a secondary analysis of a previously completed clinical trial that is registered at clinicaltrials.gov (NCT01024816) on December 3, 2009.

**Supplementary Information:**

The online version contains supplementary material available at 10.1186/s40364-021-00317-5.

## Background

Type 2 diabetes (T2DM) affects more than 460 million individuals globally [1] and is associated with $245 billion in costs annually in the United States alone. [2] Individuals with T2DM are at risk for a number of serious complications, including cardiovascular diseases, retinopathy, and renal disease. [2] One of the primary challenges to preventing and treating T2DM is the incomplete understanding of its multifactorial etiology. [3] While a number of genetic risk factors for T2DM that provide some insight into the mechanisms underlying T2DM have been reported [4], modifiable lifestyle and behavioral characteristics are equally important risk factors. The growing appreciation for the large impact of this latter category of risk factors on genetic risk, broadly characterized as gene-environment interactions, has resulted in a focus on identifying and characterizing biomarkers that can reflect these complex relationships. A clearer understanding of gene-environment interactions that influence risk for T2DM may allow for better specification of risk-reduction interventions for T2DM or facilitate development of new interventions based on improved understanding of the underlying interactions.

Development of T2DM occurs on a continuum spanning normal blood glucose to prediabetes to T2DM. Fasting blood glucose (FBG) is the biomarker used to assess for risk for T2DM [5] and is easily measured in both clinical settings and a patient’s home environment. FBG can characterize glycemic variability over time, glycemic progression (e.g., from normal glucose tolerance to prediabetes or from prediabetes to T2DM), as well as glycemic improvement in response to interventions. Even prior to a T2DM diagnosis, T2DM related complications can begin to develop, making FBG an important tool for monitoring which individuals are in greatest need of interventions. The possibility for novel prodromal biomarkers that capture harmful physiological changes prior increased FBG could further improve detection of risk and prevention of T2D and related complications.

MicroRNAs (miRs) are short (i.e., 18–26 nucleotide) regulatory elements of messenger RNA translation to amino acids. Because miRs regulate gene expression, they operate as a function of both underlying genetic risk for disease as well as environmental factors such as responses to risk reduction interventions, including behavioral factors. [6, 7] Circulating miRs are easily measured from blood serum or plasma and are potential biomarkers for risk for development of T2DM, [8–10] exhibiting changes in expression levels prior to the onset of T2DM. [11, 12] There are at least two potential applications of miRs as predictive biomarkers related to T2DM: the first is improved identification of which individuals are at greatest risk for progression towards T2DM; and the second is identification of individuals who are likely to respond to risk reduction interventions, including behavioral interventions. Because miRs capture changes in physiology before the onset of elevated FBG, they have the potential to be important prodromal markers of risk for T2D.

Our prior clinical trial (i.e., Practicing Restorative Yoga vs. Stretching for the Metabolic Syndrome (PRYSMS); clinicaltrials.gov identifier NCT01024816) showed that a restorative yoga intervention was effective at decreasing FBG compared to stretching in individuals with the metabolic syndrome. [13] One of the hypothesized mechanisms underlying the beneficial effect of restorative yoga was decreased level of stress. However, a secondary analysis of the PRYSMS trial showed that cortisol levels and self-reported measures of stress decreased more in participants from the stretching control group than in the yoga intervention group. [14] Therefore, the mechanisms underlying the beneficial effects of restorative yoga on FBG remain largely unknown. Circulating miRs, by characterizing effects of behavioral interventions on underlying genetic predisposition, may be useful biomarkers of responses to and may provide new insights about the underlying mechanisms of restorative yoga.

The goal of this study was to characterize the associations between miRs and FBG both after 12-months as well as change in FBG from baseline to 12-months. To our knowledge, this is the first study to leverage a longitudinal design to determine whether miRs might be useful biomarkers to identify which individuals are likely to respond to risk reduction interventions for T2DM and which miRs might correspond with changes in FBG and therefore be potential future mechanistic targets.

## Methods

### Participants

The study sample included participants from the previously completed Practicing Restorative Yoga versus Stretching for the Metabolic Syndrome (PRYSMS) clinical trial (clinicaltrials.gov identifier NCT01024816), which tested the effects of restorative yoga versus active stretching on FBG in overweight adults at risk for T2DM. Participants in the PRYSMS study were recruited from the San Francisco and San Diego areas and met the International Diabetes Federation criteria for metabolic syndrome. [15]. Exclusion criteria from the PRYSMS trial included FBG ≥ 126 mg/dl, hemoglobin A1c (HbA1c) ≥ 7.0 %, fasting triglycerides ≥ 300 mg/dl, weight ≥ 400 pounds, neurological conditions that limited mobility, hospitalization for coronary heart disease within the past 6 months, current pregnancy or lactation, history of bariatric surgery, substance abuse, and use of medications affecting metabolic factors. Demographic and behavioral characteristics and medical history were collected by trained study personnel at the baseline visit.

### Study Design

Randomization was stratified by sex and race/ethnicity and participants were assigned to either the restorative yoga intervention or the active stretching group. Both interventions were delivered in a group setting twice weekly for the first 12-weeks, then weekly for 12-weeks, then monthly for the remainder of the trial. In addition, all participants received a presentation on healthy behaviors, including nutrition and physical activity information.

### Clinical Data Collection

Clinical data were collected at baseline, 3-, 6-, 9-, and 12-months. Participant weight was measured on a standard balance beam scale and height using a stadiometer. Waist circumference was measured using a Gullick II tape spring-tension measure at the site of maximum circumference midway between the lower ribs and the anterior superior iliac spine. The mean of two waist circumference measurements was calculated. Body mass index (BMI) was calculated as weight in kilograms divided by height in meters squared.

FBG was measured using an automated analyzer with an immobilized enzyme biosensor (YSI 2300 STAT Plus, YSI Life Sciences, Yellow Sprints, OH). Total cholesterol, triglycerides and HDL-cholesterol were measured by enzymatic calorimetric methods (Quest Diagnostics, San Jose, CA), and LDL-cholesterol was calculated using the Friedewald equation [16]. Blood used for banking of plasma was collected by venipuncture into vacutainers containing the preservative EDTA, centrifuged at 4 °C to separate plasma from cellular blood components, and stored at -80 °C.

### Molecular Data Collection

Plasma specimens were banked at the same time points as when clinical data were obtained (i.e., baseline, 3-months, 6-months, 9-months, and 12-months) for the parent trial. The Firefly Bioworks Multiplex Circulating MicroRNA Assay (Abcam, MA) was used for direct quantification of miRs from plasma at all five timepoints. MiRs were hybridized to complementary oligonucleotides covalently attached to encoded hydrogel microparticles. The bound target was ligated to oligonucleotide adapter sequences that serve as universal PCR priming sites. The miR-adapter hybrid models were then denatured from the particles and reverse transcription polymerase chain reaction (RT-PCR) was performed using a fluorescent forward primer. Once amplified, the fluorescent target was rehybridized to the original capture particles and scanned on an EMD Millipore Guava 6HT flow cytometer (Merck KGaA Darmstadt, Germany). Expression levels of 59 miRs (Supplemental Table 1) were measured from plasma specimens collected at each of the five timepoints on a subset of 86 participants from the PRYSMS trial. The selection of these 59 miRs was based on a previous discovery analysis from a larger set of miRs in an independent subset of participants from the PRYSMS trial (*manuscript in press*). In this discovery analysis, expression levels of 336 reliably detectable circulating miRs were measured on a subset of 10 participants in 2016 and 402 reliably detectable circulating miRs were measured from an additional subset of 10 participants in 2018. The 59 samples included in the study described in this manuscript represent the union of all miRs that were significantly differentially expressed between individuals with normal and stable FBG levels compared to those with elevated and highly variable FBG over the 12-month trial period in the discovery analyses. All miRs and all sample wells included in this experiment passed quality control measures, which included a signal > 1,000 arbitrary units or detection of a spike-in control and a blank signal from particles with no microRNA probes.

The study described here included the subset of participants with banked plasma specimens from the baseline study visit with availability of at least two additional follow-up timepoints (n = 82). Samples were not previously thawed.

### Statistical Analysis

Descriptive statistics were calculated to examine and evaluate the demographic and clinical characteristics of participants. (R, 2019) Means and standard deviations are reported for continuous variables and counts and percentages are reported for categorical variables. Comparisons between the intervention and control groups used independent groups Student’s t-test for continuous variables and chi-squared tests for categorical variables. Missing values for FBG were imputed using the mean FBG for the individual participant.

The absolute number and range of copies of individual miR that occur in a biological sample, as estimated using the flow cytometry-based Firefly assay in arbitrary units (AU), can vary considerably. To control for the differences in the scale of the miRs measured by AUs, miRs were standardized using z-scores to facilitate cross-miR comparisons of estimates of association with FBG measures.

A limitation of approaches to adjustment for multiple comparisons is that they may not be appropriate given the underlying assumption that all tests are independent, whereas the roles of individual miRs associated with a given physiological process are likely not independent. In this study, we applied a data-driven approach to determine which combination of miRs (of all possible combinations of the 59 measured miRs) accounted for the largest estimated proportion of the variability (i.e., R^2^ value) for both FBG after 12-months and change in FBG from baseline to 12-months. MiRs were used in linear models alone and then with adjustment for statistically relevant covariates. Models were fit overall and by intervention group. For the analyses stratified by group, *p* < 0.10 was used as a criterion for selection in order to identify miR predictors that might otherwise be missed because of multicollinearity. [17] Estimates of association and statistical test of significance for each covariate and the overall models are reported. All statistical modeling was done using Stata version 16.1, College Station, TX.

Expression of individual miRs was normalized using the set of miR probes (i.e., hsa-miR-92a-3p, hsa-mir-93-5p, hsa-miR-17-5p) identified by the geNorm algorithm for each experiment. [18] All included miR targets passed quality control measures and were retained in the analysis.

For the miRs that were included in the models, box and whisker plots were created in order to assess the overall variability in individual miRs (Microsoft Excel 2019, Redmond WA).

The TargetScan database was used to identify predicted messenger RNA (mRNA) targets of the miRs that were identified as optimal predictors of FBG and identify which miRs are within families. [19]

## Results

### Participant Characteristics

A total of 82 participants from the PRYSMS trial were included in this analysis. The mean age was 55 ± 7 years, the majority identified as female (73 %) and White (70 %) and had a college degree or higher level of education (66 %). The study sample was obese with a mean BMI of 35.1 ± 7.2 kg/m^2^. Overall, the group met the criteria for prediabetes with a mean FBG of 104 ± 13 mg/dL. Additional demographic and clinical characteristics in the overall sample and by intervention group are shown in Table 1 and Supplemental Tables 2 and 3. In this subset of 82 participants from the PRYSMS trial, FBG at baseline was 105 ± 13 mg/dL in the intervention group compared to 100 ± 11 mg/dL in the control group (*p* = 0.08). The change in FBG over 12-months was − 4 ± 10 mg/dL in the intervention group compared to 9 + 40 mg/dL the control group (*p* < 0.05).

**Table. 1:** Demographic and Clinical Characteristics of the Sample

n (%) oraverage ± standard deviation	*n* = 82
**Age (years)**	55 ± 7
**Male sex (n)**	22 (27)
**Completed College**	54 (66)
**Race**	
** Asian**	11 (13)
** Black**	4 (5)
** Latin**	8 (10)
** White**	57 (70)
** Other/Mixed**	2 (2)
**Glucose (serum) (mg/dL)**	104 ± 13
**Total Cholesterol (mg/dL)**	206 ± 39
**Triglycerides (mg/dL)**	167 ± 63
**LDL Cholesterol (mg/dL)**	125 ± 35
**HDL Cholesterol (mg/dL)**	49 ± 11
**Body Mass Index (kg/m** ^**2**^ **)**	35.1 ± 7.2
**Waist Circumference (cm)**	110 ± 13
**Hip Circumference (cm)**	117 ± 12
**Weight (pounds)**	211 ± 43
**Systolic Blood Pressure (mmHg)**	124 ± 15
**Diastolic Blood Pressure (mmHg)**	72 ± 8

*cm* centimeters; *dL* deciliters; *HDL* high density lipoprotein; *kg* kilograms; *LDL* low density lipoprotein; *m* meters; *mg* milligrams; *mmHg* millimeters of mercury

### Associations Between miRs and FBG

Of the 59 measured miRs, none were independently associated with FBG after 12-months or the change in FBG after 12-months in univariate models.

Using a general linear model, 14 miRs (let-7c, miR-17, miR-20b, miR-22, miR-92a, miR-93, miR-106b, miR-167d, miR-192, miR-197, miR-296, miR-342, miR-363, miR-374b) were identified as the optimal set of miR predictors of FBG at 12-months with an overall model R-squared of 0.754 (*p* < 0.001) (Table 2). Addition of the three covariates that differed between the intervention and control group (i.e., waist and hip circumference, weight) did not change the observed differences, nor were these variables significant in the overall model. For change in FBG after 12-months, 12 miRs (let-7c, miR-17, miR-20b, miR-22, miR-92a, miR-93, miR-106b, miR-186, miR-192, miR-296, miR-342, miR-374b) were identified as the optimal set of predictors with an overall model R-squared of 0.731 (*p* < 0.001) (Table 2). To facilitate interpretation of single unit changes in miR expression (in AU) to single unit change in FBG (mg/dL), non-standardized values for individual miRs that were significant predictors in the models are shown in Table 3.

**Table 2:** Standardized^a^ MicroRNA Predictors of Fasting Blood Glucose

	Fasting Blood Glucoseat 12-Months			Change in Fasting Blood Glucose after 12-Months		
***R***^**2**^**(*p*-value)**	**0.754 (*p* < 0.001)**			**0.731 (*p* < 0.001)**		
	**β**	**SE**	***p*-value**	**β**	**SE**	***p*-value**
(Constant)	116.835	9.455	< 0.001			
let-7c-5p	57.793	17.213	**0.006**	36.473	15.538	**0.039**
miR-17-5p	103.369	56.573	0.095	115.762	51.070	**0.045**
miR-20b-5p	-44.197	19.784	**0.047**	-46.590	17.859	**0.024**
miR-22-3p	54.453	21.730	**0.029**	40.900	19.616	0.061
miR-92a-3p	153.475	75.753	0.068	155.501	68.383	**0.044**
miR-93-5p	109.430	47.467	**0.042**	102.078	42.849	**0.036**
miR-106b-5p	-36.680	12.263	**0.012**	-26.795	11.070	**0.034**
mir-186-5p	-10.961	22.123	0.283	-12.247	23.123	0.456
miR-192-5p	-57.402	21.394	**0.021**	-41.995	19.312	0.052
miR-197-3p	82.176	32.636	**0.029**			
miR-296-5p	42.881	13.913	**0.010**	35.448	12.560	**0.017**
miR-342-3p	29.558	11.615	**0.027**	25.671	10.485	**0.032**
miR-363-3p	24.191	11.045	0.051			
miR-374b-5p	40.072	22.111	0.097	36.522	19.960	0.094

^a^Because expression levels measured by flow cytometry cannot be directly compared between individual microRNAs, all microRNA values were standardized to z-scores so that the mean expression level is equal to zero and a 1-unit change is equal to one standard deviation from the mean

*β* beta value; *SE* standard error

**Table 3:** Non-standardized microRNA values associated with fasting blood glucose outcomes

	Final Fasting Blood Glucoseat 12-Months			Change in Fasting Blood Glucose after 12-Months		
***R***^**2**^**(*p*-value)**	**0.873 (*p* < 0.001)**			**0.734 (*p* < 0.001)**		
	**β**	**SE**	***p*-value**	**β**	**SE**	***p*-value**
miR-106b-5p	-0.007	0.009	0.431	-0.010	0.013	0.431
let7c-5p	-0.003	0.009	0.763	-0.007	0.015	0.659
miR-20b-5p	-0.003	0.004	0.529	0.003	0.006	0.664
miR-296-5p	-0.184	0.091	**0.045**	-0.237	0.150	0.120
miR-342-3p	0.020	0.017	0.257	0.068	0.028	**0.019**
miR-92a-3p	0.023	0.003	**0.000**	0.022	0.003	**0.000**
mir-93-5p	0.021	0.005	**0.000**	0.021	0.004	**0.000**
miR-17-5p	0.016	0.004	**0.000**	0.014	0.003	**0.000**

β-values represent single unit change in flow cytometry-measured fluorescence signal (arbitrary units (AU)) that corresponds to a single unit change in FBG (mg/dL)

*β* beta; *FBG* fasting blood glucose; *SE* standard error

There was no statistically significant interaction with intervention group for FBG at 12-months or change in FBG over 12 months. However, in stratified analyses by intervention group, there were some differences in the subset of miRs identified as the optimal predictors of FBG. The active control group retained five miRs that were independently statistically significant (i.e., let-7c, miR-92a, miR-93, miR-363, miR-374b), whereas the intervention group retained only two miRs (miR-92a, miR-93) with independent statistical significance (*p* < 0.1 for all). These associations were the same for both FBG outcomes (i.e., at 12 months, change from baseline at 12 months) (Supplemental Tables 4 and 5).

The full list of all predicted mRNA targets of miRs identified in the models is shown in Supplemental Table 6. There are a total of 11,584 predicted miR-mRNA pairings. Some of the identified miRs in both predictive models are within families. There two sets of families identified. The first includes miR-17-5p, miR-20b-5p, miR-93-5p, and miR-106-5p. The second includes miR-92a-3p and miR-363-3p.

## Discussion

This study assessed whether microRNAs were significant predictors of FBG after a 12-month behavioral intervention in participants with prediabetes. Using linear modelling, we assessed both FBG at 12 months and the change in FBG after 12 months and identified 14 and 12 miRs that were statistically significant predictors, respectively. The models overall accounted for significant variability in both FBG outcomes (i.e., R^2^ > 0.7). While no evidence of statistical interaction by intervention group was observed, three miRs were significant predictors of FBG in the active control group but not the intervention group, suggesting the effects of the intervention on FBG may be mediated by miRs. Finally, we assessed the variability of the significant miR predictors from the model for FBG after 12 months and identified miRs with the greatest variability and potential for a greater physiological range.

Prior studies have typically focused on the associations of single miRs with T2DM and related outcomes based on *a priori* hypotheses or agnostic hypothesis-generating approaches with correction for multiple comparisons. In contrast, we sought to use linear modelling to derive the set of miRs that was the most accurate for prediction of FBG outcomes. There is a growing consensus that miRs act synergistically through co-regulation of individual genes and sets of genes that have related functions or are co-located in biological pathways. [20] A limitation of the agnostic approach with adjustment for multiple comparisons is that it may not be appropriate given the underlying assumption that all tests are independent, whereas the roles of individual miRs associated with a given physiological process are likely not independent. In this study, we applied a data-driven approach to determine which combination of miRs accounted of the largest estimated proportion of the variability for both FBG after 12-months and change in FBG from baseline to 12-months. This approach not only allows for greater statistical power by including a set of miR predictors, but also offers the possibility of identifying sets of miRs that may co-regulate genes, sets of genes, and biological pathways that underlie risk for T2DM. For example, both miR-192 and miR-20b target components of peroxisome proliferator activated receptor gamma (PPARG) [21], which is located in the Type II diabetes mellitus pathway [22]. PPARG is primarily expressed in adipose tissue, has a role in regulation of adipocyte differentiation, and has been associated with risk for obesity and T2DM. [23] In addition, this receptor is the target of the thiazolidenidone class of medications that are used to improve insulin sensitivity and decrease risk for T2DM or control FBG in individuals diagnosed with T2DM. *In silico* analysis of the predicted mRNA and biological pathway targets of the sets of miRs associated with FBG may provide additional information about larger networks that underlie risk for T2DM. Further, the miRs identified by these models are potential therapeutic targets that may be able to impact multiple mechanisms underlying risk for T2DM.

For most miRs, the complete biological implications are not fully known. This study validated a subset of the findings observed in our preliminary discovery analysis of an independent subgroup of participants from the PRYSMS study sample. For example, in an independent sample, miR-192, miR-93, and miR-197 were previously found to be associated with variability (i.e., individuals showing high variability in FBG compared to individuals with stable FBG) over 12-months. We also previously observed cross-sectional associations between miR-106b and miR-22 and baseline FBG. [24] In another study sample, we observed associations between miR-197 and glycemic progression over 12-months [12] and let-7c and 106b showed changes in expression following a 3-month behavioral intervention for weight loss [24]. There is evidence that miR-93 has a role in processes related to T2DM, including insulin resistance and subsequent polycystic ovarian syndrome [25], and a prior genome-wide association study found an association between insulin like growth factor binding protein 5 (*IGFBP5*), which is a mRNA target of miR-197, and adipose tissue volume in women. [26] Our own recent *in silico* analyses of the 57 miRs evaluated here showed clear enrichment of target genes on pathways relevant to T2DM. For example, *AKT1* is a mRNA target of miR-192. *AKT1* is highly expressed in the pancreas and liver, both of which are related to the pathophysiology of T2DM, including skeletal muscle uptake of glucose, glucose metabolism and lipogenesis in adipose tissue, and hepatic glucose production. [27] Multiple studies showed decreased expression of miR-106b following bariatric surgery. [28] MiR-106b is associated with GLUT4 expression and translocation in response to insulin-induced glucose uptake in skeletal muscle. [29] *In vitro* studies of human adipocyte differentiation showed consistent changes in expression of miR-106b in mature adipocytes compared to their precursors, and miR-106b targets genes located in pathways associated with adipocyte differentiation. [30]

For the remaining miRs associated with FBG, the biological functions are less clear, but associations with T2DM and related conditions have been reported. MiR let-7c is as a potential therapeutic target to mediate the activity of transforming growth factor β1 (*TGF-*β*1*) in diabetic kidney disease and [31] and polarization of macrophages in immune responses [32]. MiR-186, [33] miR-342, [34] miR-363, [35] miR-374, [36] and miR-92a [37] have been identified in studies of vascular endothelial cell function and therefore may be more related to complications from, rather than the etiology of, T2DM. Similarly, miR-296 appears to target sodium-glucose transporter-2 (*SGLT2*) to promote wound healing in individuals with T2DM [38]. Additional studies exploring the functional role of these miRs related to T2DM and whether they can serve as predictive markers of not only T2DM but also related complications are needed.

**Fig. 1:**
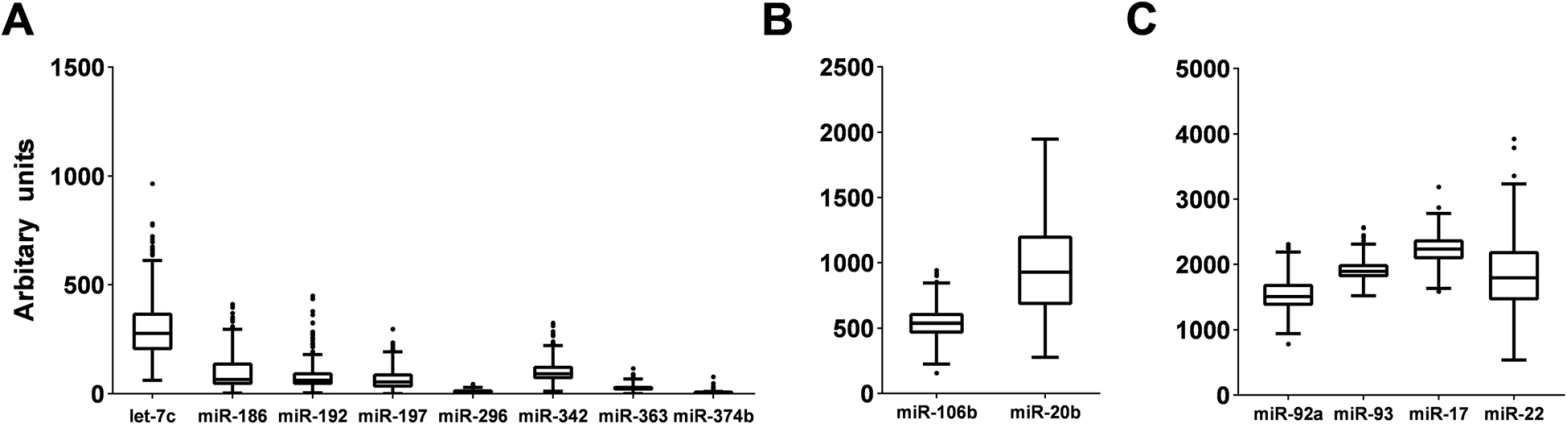
Box and Whisker Plots Depicting Variability in MicroRNA Expression Levels. Within each box, the bottom border represents the 25th percentile, the center line represents the 50th percentile, and the upper border represents the 75th percentile. The lowest horizontal line represents the minimum, and the upper horizontal line represents the maximum. Small black circles represent outliers. Individual microRNAs are represented on the x-axis. Arbitrary florescence units (AU) are represented on the y-axis. Because the overall range of AU was large, subsets of microRNAs were grouped into panels. Panel A shows microRNAs with median AUs < 500. Panel B shows microRNAs with AUs between 500–1000. Panel C shows microRNAs with AUs > 1000

MiRs with narrow physiological ranges (i.e., low variability in expression levels) may be difficult to manipulate for therapeutic purposes and result in off-target effects. Conversely, manipulation of miRs that show a greater dynamic range may be better tolerated and more variable miRs may be better therapeutic targets. Therapeutic manipulation of miRs can occur through administration of synthetic miR mimics or inhibitors that bind to endogenous miRs and prevent binding to their mRNA targets. The variances of the miRs included in models for FBG after 12-months are shown in Fig. 1. Let-7c, miR-20b, and miR-22 show relatively greater variance compared to others. Preliminary evidence supports associations between miR-20b and risk for T2DM, including insulin uptake in skeletal muscle [39] and T2DM-related complications including diabetic retinopathy [40] and endothelial cell function [41]. Similarly, miR-22 has been associated with adipose tissue mass, insulin sensitivity, and glucose homeostasis [42] and diabetic cardiomyopathy [43]. Evaluation of which miRs have a high relative variance and show evidence for associations with risk for T2DM may prioritize which miRs may be therapeutic targets controlling or decreasing FBG and preventing onset of T2DM.

Three microRNAs (i.e., let-7c, miR-363, miR-374b) were significant contributors to the models for both final FBG and change in FBG in the active control group but not the restorative yoga intervention group (Table 4). This finding may provide some insights into the potential mechanisms underlying the effect of restorative yoga on FBG observed in the PRYSMS trial. [13] A prior secondary analysis of the PRYSMS trial investigated the hypothesis that the effect of restorative yoga may be through decreased stress. However measures of both perceived stress and salivary cortisol failed to support this hypothesis. [14] MiR-363 and miR-374b have largely been studied in relation to cancer. However, miR-374b downregulates expression of phosphatase and tensin homologue (PTEN), which is associated with impaired insulin sensitivity, a major risk factor for T2DM. [44] An animal model study showed changes in spermatozoic let-7c levels in response to a high fat diet that were maintained in offspring and that let-7c targets mRNAs expressed in white adipose tissue. [45] The lack of association of these miRs with FBG outcomes in the intervention group may indicate that restorative yoga decreases expression of these miRs in individuals who are at risk for T2DM with potential downstream upregulation of their mRNA targets and corresponding protein products. Additional studies, including *in silico* analysis, of the predicted mRNA and biological pathway targets of the miRs that were associated with stretching but not yoga in individuals at risk for T2DM are needed.

**Table. 4:** Overlapping MicroRNAs By Intervention Group and Blood Glucose Outcome

		Final FBG		Change in FBG	
		**C**	**I**	**C**	**I**
**MicroRNAs**	**let-7c**	X		X	
	**miR-363**	X		X	
	**miR-374b**	X		X	
	**miR-92a**	X	X	X	X
	**miR-93**	X	X	X	X

*C* control group; *FBG* fasting blood glucose; *I* intervention group

## Conclusions

This study focused on circulating miRs that predicted FBG at 12-months as well as change in FBG after 12-months. This is one of the first studies to use a longitudinal design to assess whether miRs can predict changes in FBG over time. Rather than evaluating single miRs as predictors, we used linear modelling to identify the optimal subsets of miRs, which resulted in approximately 75 % of the variance in FBG outcomes explained by the models. Among the identified miRs, several have previously been associated with risk for T2DM in other studies, and preliminary evidence suggests that others could be related to vascular endothelial complications from T2DM. Finally, we identified three miRs that were included in the set of optimal predictors for the active control group but not the intervention group. Further exploration of the function of these miRs may provide new insights about the poorly understood positive impact of restorative yoga on FBG. This study had limitations. Not all participants in the PRYSMS dataset had sufficient banked biospecimens available for inclusion in the current study, thus the sample may not be representative of the full PRYSMS study sample. However, as with the full PRYSMS sample, there were no significant differences in FBG at baseline between the intervention and control groups. We previously performed a discovery analysis using an independent sample of participants from the PRYSMS trial that measured the subset of reliably detectable circulating miRs in order to inform the selection of the 59 miR included in the design of the custom assay used in the study described in this paper. Additional relevant miRs may have been excluded in the custom assay. Future studies that are powered to include a larger number of miRs may identify these additional relevant miRs as useful additions to prediction models. Future directions include replication of these findings in an independent sample to determine whether the prediction models still explain a high level of variance in FBG and functional assessment of the miRs included in the models to further understand their potential mechanistic contributions to risk for T2DM and responses to behavioral risk reduction interventions.

## Supplementary Information

**Additional file 1:**

**Additional file 2:**

**Additional file 3:**

**Additional file 4:**

**Additional file 5:**

**Additional file 6:**

## Data Availability

The datasets generated and/or analyzed during the current study are not publicly available as there are no public repositories for this type of dataset. The data are available from the corresponding author on reasonable request.

## Abbreviations

BMI: Body mass index
FBG: Fasting blood glucose
miR: microRNA
