# Supplementary material for: Circulating MicroRNAs predict glycemic improvement and response to a behavioral intervention
Source: Biomark Res. 2021 Aug 23;9:65. doi: 10.1186/s40364-021-00317-5 (PMC8383422; doi:10.1186/s40364-021-00317-5)
Supplement: Supplementary file 1 — Additional file 1: [file 40364_2021_317_MOESM1_ESM.docx]

**Supplemental Table 1.** MicroRNAs Included in the Firefly Bioworks Multiplex Circulating MicroRNA Assay

| **MicroRNA Name** |
| --- |
| hsa_let_7c_5p |
| hsa_let_7f_5p |
| hsa_mir_106b_5p |
| hsa_mir_126_3p |
| hsa_mir_126_5p |
| hsa_mir_1287_5p |
| hsa_mir_130b_3p |
| hsa_mir_133b |
| hsa_mir_136_3p |
| hsa_mir_140_3p |
| hsa_mir_144_3p |
| hsa_mir_145_5p |
| hsa_mir_151a_3p |
| hsa_mir_151a_5p |
| hsa_mir_151b |
| hsa_mir_181c_3p |
| hsa_mir_186_5p |
| hsa_mir_192_5p |
| hsa_mir_197_3p |
| hsa_mir_197_5p |
| hsa_mir_203a_3p |
| hsa_mir_205_5p |
| hsa_mir_206 |
| hsa_mir_20b_5p |
| hsa_mir_215_5p |
| hsa_mir_221_3p |
| hsa_mir_23a_3p |
| hsa_mir_23b_5p |
| hsa_mir_24_3p |
| hsa_mir_27a_3p |
| hsa_mir_296_5p |
| hsa_mir_29b_3p |
| hsa_mir_30a_5p |
| hsa_mir_320c |
| hsa_mir_323a_3p |
| hsa_mir_326 |
| hsa_mir_330_3p |
| hsa_mir_342_3p |
| hsa_mir_342_5p |
| hsa_mir_345_5p |
| hsa_mir_363_3p |
| hsa_mir_374b_5p |
| hsa_mir_379_5p |
| hsa_mir_422a |
| hsa_mir_424_5p |
| hsa_mir_425_3p |
| hsa_mir_486_3p |
| hsa_mir_503_5p |
| hsa_mir_532_5p |
| hsa_mir_652_3p |
| hsa_mir_874_3p |
| hsa_mir_877_5p |
| hsa_mir_92a_3p |
| hsa_mir_93_5p |
| hsa_mir_98_5p |
| hsa_mir_15b_5p |
| hsa_mir_16_5p |
| hsa_mir_17_5p |
| hsa_mir_22_3p |
