# Supplementary material for: Circulating MicroRNAs predict glycemic improvement and response to a behavioral intervention
Source: Biomark Res. 2021 Aug 23;9:65. doi: 10.1186/s40364-021-00317-5 (PMC8383422; doi:10.1186/s40364-021-00317-5)
Supplement: Supplementary file 2 — Additional file 2: [file 40364_2021_317_MOESM2_ESM.docx]

**Supplemental Table 2.** Demographic and Clinical Characteristics by Intervention Group

| % (n) or  average ± standard deviation | Control  (n=31) | Intervention  (n=51) |  | p-value |
| --- | --- | --- | --- | --- |
| Age (years) | 53.9 ± 7.4 | 55.5 ± 6.3 |  | 0.318 |
| Male sex (n) | 35.5 (11) | 21.6 (11) |  | 0.203 |
| Completed College | 64.5 (20) | 66.7 (34) |  | 1.000 |
| Race |  |  |  | 0.951 |
| Asian | 12.9 (4) | 13.7 (7) |  |  |
| Black | 3.2 (1) | 5.9 (3) |  |  |
| Latin | 9.7 (3) | 9.8 (5) |  |  |
| White | 74.2 (23) | 66.7 (34) |  |  |
| Other/Mixed | 0(0) | 3.9 (2) |  |  |
| Glucose (Serum) (mg/dL) | 100.6 ± 10.8 | 105.4 ± 13.4 |  | 0.078 |
| Total Cholesterol (mg/dL) | 207.1 ± 39.9 | 205.2 ± 38.9 |  | 0.834 |
| Triglycerides (mg/dL) | 172.1 ± 78.5 | 163.3 ± 51.6 |  | 0.582 |
| LDL Cholesterol (mg/dL) | 122.7 ± 36.7 | 126 ± 34.3 |  | 0.686 |
| HDL Cholesterol (mg/dL) | 50 ± 11.4 | 48.1 ± 11 |  | 0.464 |
| Waist Circumference (cm) | 104.8 ± 13.6 | 112.7 ± 11.7 |  | 0.010 |
| Hip circumference (cm) | 109.6 ± 9.1 | 121.1 ± 12.2 |  | <0.001 |
| Weight (pounds) | 197.4 ± 47.1 | 218.8 ± 37.8 |  | 0.036 |
| Body Mass Index | 31.1 ± 5.0 | 37.6 ± 7.3 |  | <0.001 |
| Systolic Blood Pressure (mmHg) | 124.8 ± 15 | 124.2 ± 15.3 |  | 0.855 |
| Diastolic Blood Pressure (mmHg) | 73.8 ± 8.2 | 70.8 ± 8.3 |  | 0.119 |

cm – centimeters; dL – deciliters; HDL – high density lipoprotein; LDL – low density lipoprotein; mg – milligrams; mmHg – millimeters of mercury
