# Supplementary material for: Circulating MicroRNAs predict glycemic improvement and response to a behavioral intervention
Source: Biomark Res. 2021 Aug 23;9:65. doi: 10.1186/s40364-021-00317-5 (PMC8383422; doi:10.1186/s40364-021-00317-5)
Supplement: Supplementary file 3 — Additional file 3: [file 40364_2021_317_MOESM3_ESM.docx]

**Supplemental Table 3.** Demographic and Clinical Characteristics at 12-months

| % (n) or  average ± standard deviation | Overall  (n=77) |
| --- | --- |
| Male Sex (n) | 21 (27.3) |
| BS Degree | 49 (63.6) |
| Race |  |
| Asian | 10 (13) |
| Black | 4 (5.2) |
| Latin | 10 (13) |
| White | 52 (67.5) |
| Other/Mixed | 1 (1.3) |
| Glucose (Serum) (mg/dL) | 105.48 ± 26.43 |
| Total Cholesterol (mg/dL) | 199.96 ± 32.93 |
| Triglycerides (mg/dL) | 170.57 ± 92.37 |
| LDL Cholesterol (mg/dL) | 119.29 ± 30.76 |
| HDL Cholesterol (mg/dL) | 48.84 ± 12.65 |
| Waist Circumference (cm) | 108.55 ± 13.70 |
| Weight (lbs.) | 210.65 ± 45.08 |
| Hip circumference (cm) | 116.60 ± 12.94 |
| Average Systolic (mm Hg) | 126.16 ± 15.26 |
| Average diastolic (mm Hg) | 70.16 ± 10.06 |
| Age (years) | 54.73 ± 6.84 |
| BMI (kg/m^2^) | 35.4 ± 7.3 |
