# Supplementary material for: Circulating MicroRNAs predict glycemic improvement and response to a behavioral intervention
Source: Biomark Res. 2021 Aug 23;9:65. doi: 10.1186/s40364-021-00317-5 (PMC8383422; doi:10.1186/s40364-021-00317-5)
Supplement: Supplementary file 4 — Additional file 4: [file 40364_2021_317_MOESM4_ESM.docx]

**Supplemental Table 3.** MicroRNA Predictors of Fasting Blood Glucose after 12-months by Intervention Group

|  | **Control Group** | | | **Intervention Group** | | |
| --- | --- | --- | --- | --- | --- | --- |
| **R^2^ (p-value)** | **0.754 (p<0.001)** | | | **0.257 (p<0.001)** | | |
|  | **β** | **SE** | **p-value** | **β** | **SE** | **p-value** |
| (Constant) | 104.332 | 4.786 | <0.001 | 107.550 | 6.655 | <0.001 |
| let-7c-5p | 28.223 | 7.288 | **0.002** | 14.453 | 10.739 | 0.188 |
| miR-17-5p | -63.481 | 41.117 | 0.147 | 59.777 | 41.046 | 0.155 |
| miR-20b-5p | 1.275 | 6.707 | 0.852 | -10.043 | 10.104 | 0.328 |
| miR-22-3p | 3.265 | 3.508 | 0.369 | -3.548 | 8.989 | 0.696 |
| miR-92a-3p | -91.735 | 49.144 | **0.085** | 100.494 | 59.073 | **0.099** |
| miR-93-5p | -56.666 | 29.612 | **0.078** | 58.357 | 33.350 | **0.090** |
| miR-106b-5p | -5.758 | 5.573 | 0.320 | -12.790 | 7.982 | 0.119 |
| mir-186-5p | 0.470 | 2.590 | 0.859 | -7.265 | 9.464 | 0.448 |
| miR-192-5p | -5.593 | 4.337 | 0.220 | 0.257 | 8.994 | 0.977 |
| miR-197-3p | 6.135 | 7.290 | 0.415 | 2.716 | 8.684 | 0.757 |
| miR-296-5p | 0.596 | 4.798 | 0.903 | 7.072 | 7.088 | 0.326 |
| miR-342-3p | 6.297 | 3.852 | 0.126 | 8.277 | 7.782 | 0.296 |
| miR-363-3p | -9.094 | 4.231 | **0.051** | 14.463 | 9.080 | 0.121 |
| miR-374b-5p | -8.247 | 3.482 | **0.034** | -5.542 | 10.960 | 0.617 |

Because expression levels measured by flow cytometry cannot be directly compared between individual microRNAs, all microRNA values were standardized to z-scores so that the mean expression level is equal to zero and a 1-unit change is equal to one standard deviation from the mean.

β – Beta value; SE – standard error
