# Supplementary material for: Circulating MicroRNAs predict glycemic improvement and response to a behavioral intervention
Source: Biomark Res. 2021 Aug 23;9:65. doi: 10.1186/s40364-021-00317-5 (PMC8383422; doi:10.1186/s40364-021-00317-5)
Supplement: Supplementary file 5 — Additional file 5: [file 40364_2021_317_MOESM5_ESM.docx]

**Supplemental Table 4.** MicroRNA Predictors of Change in Fasting Blood Glucose after 12-months by Intervention Group

|  | **Control Group** | | | **Intervention Group** | | |
| --- | --- | --- | --- | --- | --- | --- |
| **R^2^ (p-value)** | **0.764 (p<0.001)** | | | **0.234 (p<0.001)** | | |
|  | **β** | **SE** | **p-value** | **β** | **SE** | **p-value** |
| (Constant) | 0.195 | 2.558 | 0.940 | 1.330 | 5.866 | 0.822 |
| let-7c-5p | 12.432 | 3.894 | **0.007** | 14.396 | 9.466 | 0.138 |
| miR-17-5p | -38.006 | 21.972 | 0.107 | 56.488 | 36.181 | 0.129 |
| miR-20b-5p | 0.602 | 3.584 | 0.869 | -8.863 | 8.907 | 0.327 |
| miR-22-3p | 1.684 | 1.875 | 0.385 | -0.456 | 7.924 | 0.954 |
| miR-92a-3p | -64.668 | 26.261 | **0.029** | 93.219 | 52.072 | **0.083** |
| miR-93-5p | -39.902 | 15.824 | **0.026** | 58.247 | 29.397 | **0.056** |
| miR-106b-5p | -0.091 | 2.978 | 0.976 | -9.124 | 7.036 | 0.204 |
| mir-167d | 0.638 | 1.384 | 0.652 | -2.148 | 8.342 | 0.798 |
| miR-192-5p | -0.893 | 2.318 | 0.706 | -0.099 | 7.928 | 0.990 |
| miR-197-3p | 3.196 | 3.896 | 0.427 | 4.097 | 7.655 | 0.596 |
| miR-296-5p | -1.361 | 2.564 | 0.604 | 7.741 | 6.248 | 0.225 |
| miR-342-3p | 0.446 | 2.059 | 0.832 | 7.508 | 6.860 | 0.282 |
| miR-363-3p | -4.030 | 2.261 | **0.098** | 8.822 | 8.004 | 0.279 |
| miR-374b-5p | -4.600 | 1.860 | **0.028** | -11.025 | 9.661 | 0.263 |

Because expression levels measured by flow cytometry cannot be directly compared between individual microRNAs, all microRNA values were standardized to z-scores so that the mean expression level is equal to zero and a 1-unit change is equal to one standard deviation from the mean.

β – Beta value; SE – standard error
